# Helminth Infections Induce Tissue Tolerance Mitigating Immunopathology but Enhancing Microbial Pathogen Susceptibility

**DOI:** 10.3389/fimmu.2018.02135

**Published:** 2018-10-16

**Authors:** George S. Yap, William C. Gause

**Affiliations:** Department of Medicine, Center for Immunity and Inflammation, Rutgers University-New Jersey Medical School, Newark, NJ, United States

**Keywords:** tolerance, helminth, resistance, confection, immune, injury, microbes

## Abstract

Helminths are ubiquitous and have chronically infected vertebrates throughout their evolution. As such helminths have likely exerted considerable selection pressure on our immune systems. The large size of multicellular helminths and their limited replicative capacity in the host necessarily elicits different host protective mechanisms than the immune response evoked by microbial pathogens such as bacteria, viruses and intracellular parasites. The cellular damage resulting from helminth migration through tissues is a major trigger of the type 2 and regulatory immune responses, which activates wound repair mechanisms that increases tissue tolerance to injury and resistance mechanisms that enhance resistance to further colonization with larval stages. While these wound healing and anti-inflammatory responses may be beneficial to the helminth infected host, they may also compromise the host's ability to mount protective immune responses to microbial pathogens. In this review we will first describe helminth-induced tolerance mechanisms that develop in specific organs including the lung and the intestine, and how adaptive immunity may contribute to these responses through differential activation of T cells in the secondary lymphoid organs. We will then integrate studies that have examined how the immune response is modulated in these specific tissues during coinfection of helminths with viruses, protozoa, and bacteria.

## Introduction

Helminths are ubiquitous and have chronically infected vertebrates throughout their evolution. A number of studies have shown that they can severely impact wild vertebrate populations affecting their body weight, fecundity and their ability to survive hardship in the winter (1–3). In humans, low-level infections can be asymptomatic, but more heavily infected individuals are adversely affected, exhibiting morbidity in adults and impaired physical and cognitive development in children (4–6). As such helminths have likely exerted considerable selection pressure on our immune systems. The large size of multicellular helminths necessarily requires different host protective mechanisms than the immune response evoked by microbial pathogens such as bacteria and viruses. Also, the immune response to microbial pathogens includes mechanisms that limit reproduction and associated dissemination of the microbe. Such controls are unnecessary with many helminths, as they need to leave the mammalian host to complete their life cycles. Instead, components of the host protective responses against helminths include coopted wound repair mechanisms, which mitigate the considerable tissue damage these parasites may cause as they traffic through vital organs such as the lung and liver (7, 8). These innate wound healing responses contribute to the type 2 immune response evoked by helminths, and provide a critical springboard for the subsequent adaptive immune response including antigen-specific effector T and B lymphocytes. This cellular damage resulting from helminth migration through tissues is a major trigger of the type 2 immune response, as danger associated molecular patterns (DAMPs) are released that induce the cytokine alarmins, IL-25, IL-33, and thymic stromal lymphopoietin (TSLP), that help drive the response. In contrast, the type 1 immune response may be more dependent on pathogen associated molecular patterns (PAMPS), where microbial structures, such as endotoxin, bind toll like receptors (TLRs) that help drive the initiation of the response, resulting in IL-12 production by myeloid cells, which in turn drives IFN-γ production by innate lymphoid cells (ILCs) and T cells. The overall protective type 2 immune response that ensues includes both resistance and tolerance mechanisms. Resistance immune mechanisms specifically impact the parasite and when effective reduce the parasite burden. Tolerance mechanisms reduce host tissue damage without affecting the parasite burden (3, 9).

The helminth induced type 2 immune response includes characteristic activation of immune cells. Although these activated immune cell lineages share stimulation of common signaling pathways, they also exhibit lineage specific activation states which support their characteristic effector functions. This type 2 immune cell activation motif was originally described in CD4+ Th2 cells, but it is now clear that this characteristic activation, also referred to as alternative activation, occurs in other T cells and also B cells, innate lymphocytes, mast cells, macrophages, basophils, eosinophils, and neutrophils (10–12). Unraveling how helminth infection differentially affects these innate and adaptive immune cells is as yet little understood and likely involves various epigenetic regulatory mechanisms. In many of these cells alternative activation is associated with the production of type 2 cytokines, including IL-4, IL-5, and IL-13, with different cell lineages preferentially expressing one or more of these cytokines. A range of other molecules are also associated with this alternative activation state including: arginase, Relmalpha, YM-1, IL-33, and several chemokines. In contrast, immune cells activated by microbial pathogens express chemokines and cytokines associated with type 1 and type 17 immunity including: IL-12, IFN-γ, IL-17, NOS2, and TNF-α. High levels of either type 1 or type 17 cytokines can result in harmful inflammation leading to tissue damage. Helminth induced immune responses also have immune regulatory components that include activation of FOXP3+ T regulatory cell responses, which can function to control harmful inflammation through their production of IL-10 (13). Although not specific to type 2 immune responses, IL-10 upregulation during helminth infection appears to have an important role in downregulating both type 1 and type 2 immunity. IL-10 independent immune regulatory effects have also been identified, which are not yet well defined (14, 15). Although the type 2 immune response has important wound healing characteristics, chronic type 2 inflammation can also be harmful, leading to fibrosis and associated tissue damage (16). It should be noted that immune regulatory cells activated during helminth infection have also been shown to inhibit chronic type 2 responses, including allergy-associated inflammation (17).

The helminth-induced type 2 immune response thus has important wound healing and anti-inflammatory properties. However, this beneficial response that helps to mediate tolerance by mitigating tissue damage during infection with these large multicellular parasites can have a dark side as well. Many properties of this immune response can potentially reduce the effectiveness of the protective response against many microbial pathogens. As coinfection with helminths and microbes affects much of the world's population, this as yet little studied area of research has considerable real world significance. In this review we will first describe helminth-induced tolerance mechanisms that develop in specific organs including the lung and the intestine, and how adaptive imunity may contribute to these responses through differential activation of T cells in the lymph nodes. With this background, we will review studies that have examined how the immune response is modulated in these specific tissues during coinfection of helminths with viruses, protozoa, and bacteria.

### Helminth-induced immune and tissue responses in the lung

Extensive and specific remodeling of tissues and associated organs can occur following invasion by specific pathogens. In turn, subsequent or even coincident coinfection by a different pathogen can markedly alter the course of the response in some cases compromising resistance and tolerance mechanisms directed against either pathogen. Recent studies have begun to unravel the mechanisms through which the intestinal nematode parasite, *Nippostrongylus brasiliensis*, influences lung tissue. As with several other intestinal nematode parasites, including human hookworms, *N. brasiliensis* larvae invade the host through skin penetration, migrate through the circulation to the lung, where they are coughed up and swallowed. Once in the intestine they mature to adults, breed and produce eggs. Following *N. brasiliensis* primary infection, the parasites enter the lung between 12 and 48 h after inoculation, and usually exit the lung 48 h later. Thus by 3–4 days after inoculation all the parasites have left the lung. This 2 day time interval in the lung triggers a cascade of immune responses that initially triggers acute lung injury (ALI), followed by rapid mitigation of lung damage, and finally subsequent chronic lung remodeling associated with fibrosis and emphysema. Understanding the immune components of this lung remodeling response has elucidated a number of tolerance mechanisms associated with the type 2 immune response.

As early as 1–2 days after *N. brasiliensis* inoculation, a pronounced increase in IL-17 triggers massive recruitment of neutrophils to the lung peaking at about 1 × 10^6^ total cells by day 2 (7). Further studies have shown that the source of IL-17 is γ/δ T cells, which are activated by chitinase-like proteins (CLPs) released by lung epithelial cells damaged by the invading larval parasites (18, 19). Thus, the CLPs are essentially acting as DAMPs triggering the initial inflammatory response. The infiltrating neutrophils contribute to ALI associated with hemorrhaging, inflammation, and impaired lung function. Mechanical damage by the helminth itself is also a factor contributing to ALI, which is pronounced by about 3 days after inoculation. The type 2 immune response, characteristic of helminth infections, becomes pronounced by day 4 and its increase coincides with a decrease in IL-17 and ALI. Blocking IL-4R signaling inhibits the development of type 2 immunity and results in sustained IL-17 elevations, neutrophil inflammation, and associated ALI (7). These studies thus demonstrated that IL-4R signaling can play an essential role in mitigating tissue damage during helminth infections.

An essential myeloid cell type activated at early stages of the type 2 immune response is the alternatively activated macrophage (AAM). As IL10 is not elevated at early stages of the response, IL-4R signaling is the major trigger and the helminth-activated macrophage also does not produce IL-10 (7, 20). However, macrophages activated through helminth infection do express a number of factors important in both control of inflammation and in directly enhancing the wound healing process. These include: insulin-like growth factor (IGF-1), Resistin-like molecule α (RELMα), and arginase 1 (Arg.1), all of which are IL-4R dependent (7, 21). RELMα and Arg. 1 have pleiotropic effects, the former being capable of both downmodulating type 2 immune responses (22, 23) and directly enhancing wound healing (24). Arg1, in addition to catalyzing arginine metabolism which results in the production of ornithine and polyamines, also can downmodulate type 1 inflammation by depleting local arginine concentrations (25). In the lung, besides IL-4Rα signaling, AAM activation and proliferation is also dependent on other factors characteristic of the lung microenvironment and the specific infectious agent. In particular, recent studies have shown that the infiltrating alternatively activated (N2) neutrophils interact with the macrophages to drive their alternatively activated phenotype. This includes both their production of IL-13 (11) and their apoptotic state which is recognized by AXL/Mertk, apoptotic sensors expressed by the macrophage (26). In addition, surfactant protein A (SPA) expressed by lung epithelial cells also drives AAM activation and proliferation (27). Thus, both myeloid cell crosstalk and the local tissue microenvironment provide critical cues driving AAM activation in the lung during helminth infection. Other myeloid and also innate lymphoid cell (ILC) populations also likely play an important role in orchestrating initiation of the type 2 immune response and mitigation of ALI. In particular, at early stages of the response ILC2 cells may provide an initial source of IL-13 and potentially other factors that drives the development of infiltrating N2 neutrophils and other components of the innate type 2 response.

**Figure 1:**
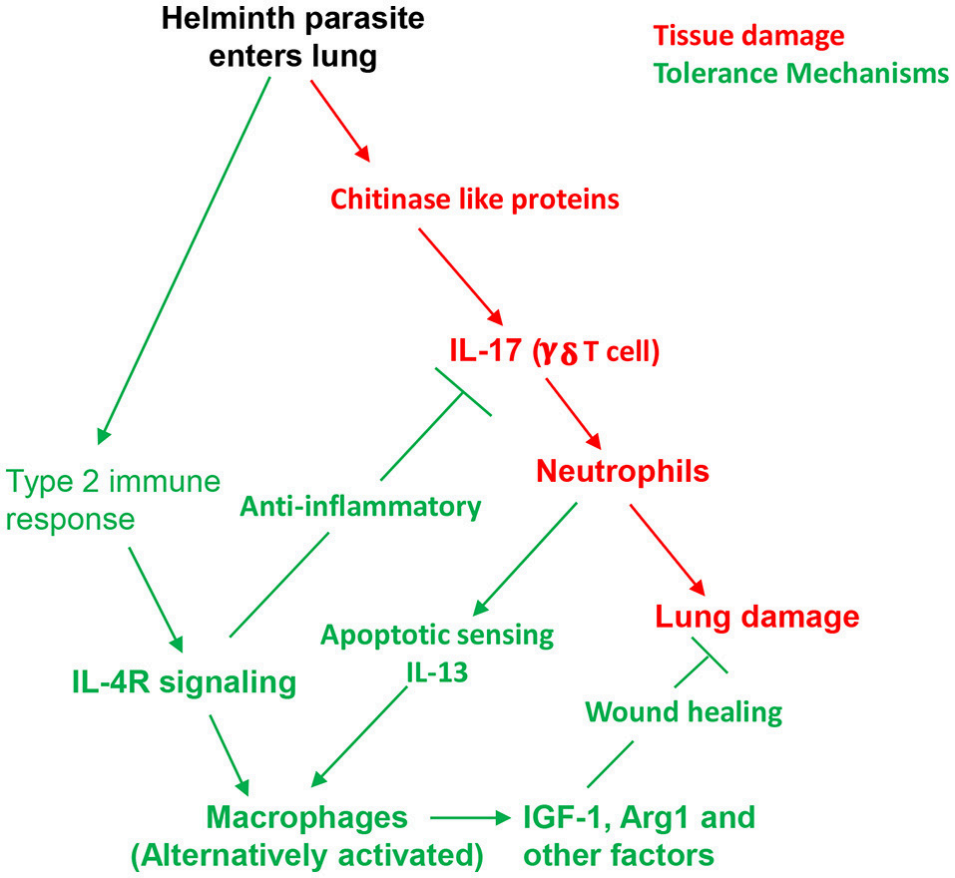
Host tolerance mechanisms contribute to protective helminth-induced type 2 response by controlling lung damage. Invasion of the lung by *N. brasiliensis* L3 triggers release of chitinase-like proteins which stimulate IL-17 production by γδ T cells and consequent recruitment of neutrophils. Inflammation and physical damage of cells by migrating helminths result in acute lung injury. Within several days a potent type 2 immune response is also induced and tolerance mechanisms dependent on IL-4R signaling inhibit IL-17. Combined signals from neutrophils, lung surfactant protein A (SPA), and direct IL-4R signaling drives alternative macrophage activation, which contributes to both anti-inflammatory and direct wound repair processes.

The above model (see Figure 1) describes helminth induced tolerance mechanisms that mitigate ALI. As a result ALI is largely resolved by 5–7 days after inoculation. However, despite the presence of the parasite in the lung for only 48 h, chronic tissue remodeling also occurs that can persist for weeks after *N. brasiliensis* inoculation. Previous studies have shown that M2 macrophages persist in the lung for at least 45 days after inoculation, and this persistent macrophage phenotype is capable of mediating acquired resistance resulting in accelerated parasite destruction upon secondary challenge (11). Also emphysema develops by day 30 after inoculation and significant fibrosis is also observed. Emphysema apparently requires infection with live parasites, as *N. brasiliensis* excretory/secretory (ES) products, which still induce type 2 responses, can drive fibrosis and associated impaired lung function, but not emphysema (28). Few studies have yet examined mechanisms contributing to emphysema following helminth infection, but secretion of proteases and elastases by myeloid cells may be causal in other emphysema models (29, 30). Intriguingly, IL-17 is also implicated in emphysema development (31, 32), raising the possibility that early elevations in IL-17 in the response to *N. brasiliensis* may trigger this specific lung tissue remodeling pathology, perhaps in part by its recruitment of neutrophils to the lung.

The type 2 immune signaling pathways activated by helminth infection in the lung may influence responses to other pathogens. The persistence of this immune milieu, as indicated by the long-lived AAM phenotype, may potentially delay or attenuate development of a type 1 immune response important in resistance against many microbial pathogens. Studies where *N. brasiliensis* inoculated mice were coinfected with *Mycobacterium tuberculosis* showed generally increased susceptibility to this intracellular bacteria, which was IL-4R dependent and transfer of WT macrophages into IL-4R^−/−^ mice restored helminth-induced susceptibility. Interestingly, however, the protective Mtb-specific Th1 cellular response was not impaired, although marked increases in AAMs were observed. Apparently, the presence of AAMs compromised effective elimination of bacteria, possibly as a result of their impaired Mtb killing and potential function as an Mtb reservoir (33). The observation that Th1 cells still developed in response to Mtb raises the intriguing possibility that type 1 and type 2 pulmonary immune responses simultaneously develop in coinfected mice and that helminth infection cannot completely override the type 1 immune response triggered by MTb, even though in this model helminth infection preceded Mtb infection by 5 days. More studies are needed to ascertain whether this apparent plasticity within the lung microenvironment is due to different separable microenvironments supporting polarized type 1 and type 2 responses or whether both polarized immune cell populations coexist in granulomas and immune cell infiltrates. Analysis of individual cells using techniques such as single cell RNAseq may also potentially reveal mixed response heterogeneity in individual myeloid cell populations.

Similar results have also recently been obtained following coinfection with the malarial parasite, *Plasmodium berghei*, and *N. brasiliensis*. In these experiments a sequential protocol was also used where *N. brasiliensis* infection preceded malarial infection by about 2 weeks. Although helminth infection blunted the protective type 1 immune response, it still was sufficiently strong to mediate effective resistance against the malarial parasite (34). In another study where mice were infected simultaneously with *N. brasiliensis* and *P. chabaudi*, type 1 immunity was not affected while type 2 cytokines were attenuated (35). However, as *N. brasiliensis* is an acute infection, with parasites residing in the host for only about 9 days, sequential or simultaneous malarial infection may not as readily modify the response as a chronic infection where the parasite persists and provides ongoing stimulation in the host. This may be in part due to plasticity in the T cell compartment, with recent studies indicating that malarial infection can rewire helminth induced Th2 cells, downmodulating their production of type 2 cytokines with a concomitant upregulation of IFN-γ (36). Also, as discussed above the initial immune response to *N. brasiliensis* is complex and includes pronounced IL-17 elevations, which may indeed be exacerbated by *Plasmodium* infections and may thus impact resistance and tolerance mechanisms. Chronic infections with other parasitic helminths including *Heligmosomoides polygyrus, Litosomoides sigmondontis*, or *Schistosoma mansoni* eventually result in a more polarized and potent type 2 immune response. In coinfection studies with these parasites, type 1 immunity and associated resistance is generally reduced, while tissue damage is mitigated (37). Thus, chronic coinfection of helminths and malarial parasites may at least in some cases impair resistance but at the same time enhance tolerance mechanisms.

### Helminth-induced immune and tissue responses in the gut

#### Initiation of the response

**Figure 2:**
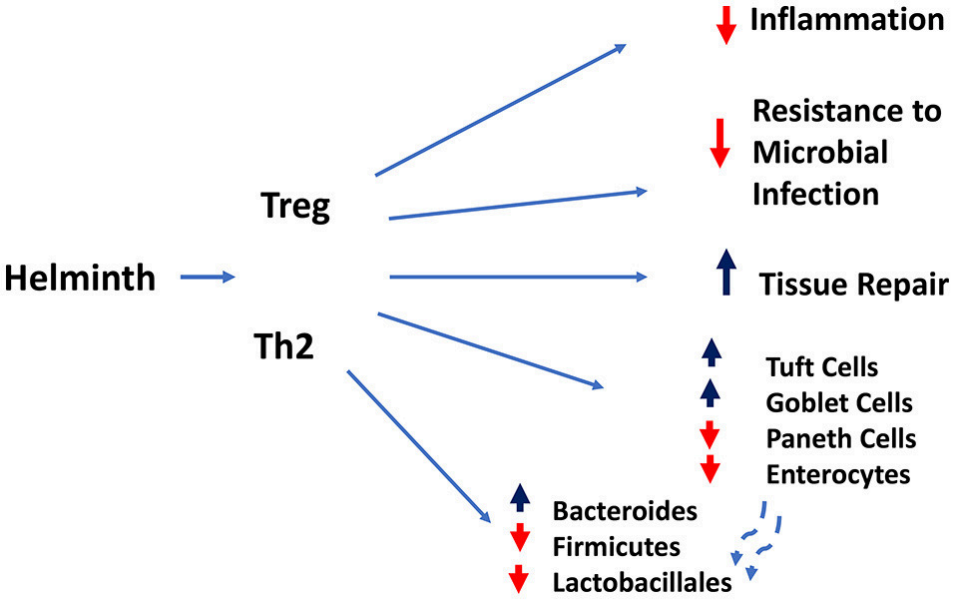
Regulatory and type 2 immune responses (mediated by T lymphocytes and other cell types) induced by helminth infection mediate dampening of inflammatory responses and compromise resistance to microbial infection, while increasing goblet and tuft cell hyperplasia and intestinal tissue repair.

The type 2 immune response triggered in the intestine by helminth infection (see Figure 2) is characterized by many of the same immune cell populations observed in lung mucosal tissues, including AAMs, differentially activated granulocytes, ILC-2s, and Th2 cells. Of course many intestinal helminths invade the skin and transit through the lungs on route to the intestine. What actually triggers the type 2 immune response in the gut is still not well understood, but it appears that it is partly triggered by endogenous danger associated molecular patterns (DAMPS) induced by tissue damage resulting from these large multicellular parasites interacting with the intestinal barrier surface.

Recent studies indicate that after infection with the murine intestinal nematode parasite *Heligmosomoides polygyrus*, adenosine interacting with the A2B adenosine receptor (A2BAR) is required for upregulation of IL-33 and the corresponding downstream type 2 immune response (38). It should be noted that the mechanism through which IL-33 works may be complex as it both binds cell surface ST2 (suppressor of tumorigenicity 2), a component of the IL-33 receptor, and also enters the nucleus as a regulatory protein (39). However, recent studies indicate that blockade of ST2 can inhibit the type 2 immune response to *H. polygyrus* (40) and that mast cells are an important source of IL-33 (41). Presumably tissue damage results in ATP release from stressed cells. The extracellular ATP is then degraded by cell surface ectonucleotidases to adenosine, which then locally accumulates extracellularly, binds cell surface A2BAR, and contributes to initiation of type 2 immunity. As such, adenosine functions as a DAMP during intestinal helminth infection alerting the host to tissue damage associated with the helminth infection (38). An epithelial cell derived molecule, trefoil factor 2 (TFF2), which can mediate tissue repair functions, has also been shown to act as a helminth-induced DAMP capable of driving initiation of the type 2 immune response through stimulation of IL-33 release (42). It will be important in future studies to investigate potential interactions and/or associations of TFF2 with A2BAR signaling as both seem to play a critical role in driving type 2 response to helminths.

Tuft cells, specialized intestinal epithelial cells, appear to recognize helminth infection through chemosensory receptor signaling. perhaps providing a mechanism for how ES products may contribute to initiation of the type 2 immune response Tuft cells are the sole producers of IL-25 in the intestine and IL-25 is essential for the type 2 immune response to H. polygyrus (43–45). Together IL-33 and IL-25 likely support the development of the innate type 2 immune response to helminths with one or the other playing a more predominant role in the development of the protective response to specific helminths. IL-33 and IL-25 can be considered cytokine alarmins, as they are the initial cytokines that signal invasion by helminths and trigger the appropriate host response. A third cytokine alarmin associated with type 2 immunity is thymic stromal lymphopoietin (TSLP), also produced by intestinal epithelial cells. In the context of helminth infection, rather than triggering type 2 immunity, TSLP appears to downregulate type 1 responses, which is particularly important in the colonic immune response to *Trichuris muris* where an underlying type 1 response, likely elicited by bacteria associated with *T. muris* invasion, is controlled by TSLP (46). In the mouse response to Schistosome infection, IL-33, IL-25, and TSLP can play partially redundant roles, with one essentially substituting for the other (47).

Helminth excretory/secretory (ES) molecules likely also contribute as inoculation with ES supernatants alone can promote a type 2 immune response (28, 48), though not as potent as a live helminth infection. The ES material derived from parasite cultures is a heterogeneous mixture composed of many bioactive molecules, ranging from small to complex glycoproteins, which are produced by the parasite to modulate the host response. They are likely a product of the dynamic relationship resulting from millions of years of vertebrate/helminth coevolution. A number of specific molecules have now been isolated from helminths and many of these can downregulate host immune responses. For example TGF-β mimic, derived from *Heligmosomoides polygyrus*, binds the TGFβ receptor and can upregulate FOXP3^+^ Treg cells through binding the TGFβ receptor (40). Also, ES62, a filarial glycoprotein, interferes with myd88 signaling, thereby inhibiting TLR (49) and IL-33 (50) signaling. Isolation of these ES products remains at a very early stage, but already potential candidates that control harmful inflammation have been identified raising the possibility that ES derived molecules could provide a rich source of future immunomodulatory therapeutics.

Thus helminth infection in both the lung and in the small intestine triggers type 2 immunity in part through tissue damage, and associated release of DAMPS, and is then further modulated through the release of helminth ES products. The type 2 immune response has many components shared with wound healing responses raising the possibility that the innate type 2 immune response may have originated from a conventional wound healing response, coopted by the immune system to mitigate tissue damage during helminth infection. The development and overlay of the adaptive type 2 immune response over the innate response may have in part evolved to incorporate antigen specificity to enhance resistance against helminths (8). Together the innate and adaptive type 2 immune response thereby mediate both tolerance and resistance mechanisms that together enhance host protection against helminths.

### Helminth-induced immunomodulation

A number of components of the helminth-induced response also directly inhibit both type 1 and type 17 responses. T regulatory (Treg) cells are expanded in response to many helminths and have been shown to downregulate harmful type 1 responses and also type 2 immunity associated with allergic responses (13). In many cases IL-10 mediates these responses, though other molecules have also been implicated, including ES products such as TGM (40). Overall, T reg cells contribute an essential component in mediating tolerance mechanisms that control inflammatory responses that would otherwise contribute to tissue damage.

The potency of the immune response evoked by helminths to downregulate harmful inflammation as well as directly promote tissue repair provides two potent and complementary tolerance mechanisms. This has direct implications on how the intestinal tissue responds to other infectious and inflammatory insults. A clear example of this concept is the finding that the gastrointestinal nematode *H. polygyrus* is able to exert potent immunomodulatory effects and inhibit intestinal inflammation induced by IL-10 deficiency (51), by TNBS hapten administration (52), and by dietary antigen challenge. As expected, multiple mechanisms appear to be induced by helminth infection including: suppression of the IL-17 response (53), activation of regulatory Foxp3+ T cells and their regulatory cytokine production (54) and the induction of “tolerogenic dendritic cells” that prevent induction of antigen specific gut T cell responses (55). Unlike the nearly uniform protective effects of helminths in inflammatory bowel disease models induced by non-viable insults, the picture that emerges from studies involving bacterial and parasitic challenges is more complex. For example, *H.polygyrus* infection exacerbates intestinal inflammation caused by *Salmonella typhymurium* infection by dampening CXCL2 chemoattraction of neutrophils, resulting in defective control of bacterial growth. In the case of challenge with the enteropathogen C*itrobacter*, where it was previously shown that bacterial burden and tissue pathology was exacerbated by H. polygyrus infection (56, 57), helminth infection induced changes in the microbiota, with increased abundance of Bacteroides and decreased representation in Firmicutes and Lactobacillales. Interestingly, gut microbiota transfer from helminth infected wildtype, but not STAT-6 deficient donors caused significant worsening of the *Cibrobacter*-induced intestinal inflammation, demonstrating an involvement of the host Th2 responses in precipitating the alterations in gut microbiota that exacerbated intestinal inflammation (58).

Strictly enteric infection with helminths can also modulate systemic immune responses. Oral Inoculation with *H. polygyrus* can control allergic responses in the lung, in part through activation of T regulatory cells (17, 59). Other immunoregulatory cell populations include B cells and macrophages, in some cases acting independently of IL-10 (48, 60). Also, oral helminth infections can control type 1 diabetes through mechanisms that involve both CD4 T cell production of IL-4 and IL-10 acting in an independent and redundant manner (61). Intriguingly excretory/secretory products derived from helminths can also have potent anti-inflammatory effects and recent studies have begun to isolate these immune modulators from a variety of helminth parasites (48, 62). Helminth infection may also perturb colonization by the intestinal microbiota thereby influencing its composition which in turn can affect immune regulation and control of harmful inflammation (63, 64). Transfer of helminth modified intestinal microbiota can protect against allergic asthma through their production of short fatty acids (SFCA) (65). In terms of coinfection, recent studies showed that *H. polygyrus* infected mice had markedly reduced pulmonary lung damage and viral load following intranasal infection with respiratory syncytial virus. The response was independent of adaptive immune responses but protection was lost in germ-free mice, indicating a role for intestinal microbiota (66). In contrast, *H. polygyrus* infection did not affect immunity or progression of disease following coinfection of mice with *Mycobacterium tuberculosis* (67). These studies indicate that although strictly enteric helminth infection may have potent systemic immunoregulatory effects, in some cases it has little effect. Understanding the conditions under which helminths can preferentially modulate a concomitant immune response will likely provide important insights into development of future therapies based on helminth treatments or on specific immune modulators purified from helminths.

Future work is needed to take into account how helminth-induced remodeling of the epithelium niches contributes to the altered tolerance of the intestine to microbial and inflammatory challenges. While it is clear that helminths induce hyperplasia of the Tuft cell and goblet cell compartments, how it impacts the absorptive epithelial and antimicrobial Paneth cell compartments remain unexplored. For example, the shift in the production of Tuft cells and Goblet cells may come at the expense of the Paneth cell niche or their ability to produce antimicrobial peptides required to maintain the normal microbiome and resist microbial challenges in the intestine. Similarly, it is not clear whether expansion of the secretory Tuft and Goblet cell niches is accompanied by a compensatory hyperplasia of the absorptive epithelial cell compartment. Future studies should also take advantage and take into account that ability of single cell (scRNASeq) technologies to resolve shifts in differentiation trajectories of intestinal stem cell and transit amplifying cells caused by helminth infection and the inherent intraniche heterogeneity and functional specialization of the Tuft cells and Goblet Cells compartments during helminth infection. A recent scRNA Seq study has already pinpointed an interesting dichotomy within the Tuft cell niche, which was previously characterized as having both neuronal and inflammatory gene expression programs. It appears that these two functional modules may be embodies in two distinct subpopulations, both of which express IL-25, IL-25 receptor (IL17rb) and receptors for IL4 and IL-13. Nevertheless, only one subset expressed high levels of TSLP and interestingly expressed CD45, a pan-marker of hemopoietic cells (68).

### Helminths alter the systemic immune landscape

As alluded to above, helminth induction of alternative immune activation and regulatory mechanisms that promote tolerance may be propagated through systemic changes in the innate and adaptive immune landscape of secondary and primary lymphoid organs. This is perhaps best exemplified by recent studies indicating that helminths induce profound shifts in the migratory behavior of both group 2 innate lymphoid cells (ILC2s) and naïve T lymphocytes (69, 70). Upon *N. brasiliensis* infection, resting ILC2s residing in the intestinal lamina propria rapidly acquire an activated KLRG1+ phenotype and become mobilized to seed the lung and liver. IL-25 appears to mediate this effect, as it is sufficient to cause activation and redistribution of activated ILCs without any apparent proliferative step. Activated ILC2s gain entry into the lymphatics and blood circulation and accumulated at distal tissue sites, including the lung, based on the well-known sphingosine-1-phosphate-mediated chemotactic mechanism used by T lymphocytes to egress from lymphoid organs. In the lung, relocalization of recently activated ILC2s promoted tissue repair and prevention of acute lung injury (69).

In contrast to the behavior of ILC2s, the naïve T and B cell pools become depleted from non-involved lymphoid organs and accumulated in the T helper 2-reactive mesenteric lymph node during helminth (*H. polygyrus*) infection in mice. This systemic redistribution of non-activated lymphocytes persists into the chronic stage of infection and requires the participation of the lymphoxin-beta receptor signaling. IL4 secretion by Th2 and Tfh cells during helminth infection likely promotes LTβ expression by follicular B cells which then expands the stromal cell compartment to reorganize lymph node architecture. Alternatively, expression of another alternate LTβR ligand, LIGHT by T cells and DCs in the reactive lymph node may be responsible for expansion of the stromal cell compartment to further promote humoral responses to the helminth parasite. Nevertheless, the relative depletion of the naïve lymphocyte pool at other lymphoid sites result in impaired responsive to heterologous immunization or infections with other unrelated micro-organisms (70).

In addition to systemic shifts in lymphocyte migration, helminth infections induces dramatic alterations in the cell type distribution and functional attributes of dendritic cells in the secondary lymphoid organs. During *N. braziliensis* infection, dermal dendritic cells acquire parasite material and migrate to the draining lymph nodes to prime CD4 T cells capable of making IL-4 (71). These dermal derived dendritic cells exhibit a unique CD11c^dull^ MHCII^hi^ phenotype and expressed Th2 promoting factors including PDL2, IRF4 and OX40L as well as CD301b (72). However, the induction of Th2-priming or Th2 associated dendritic cell types may not be sufficient for dampening opposing Th1 or Th17 responses. Recent studies indicate that IL4 exposure of dendritic cells, although resulting in the expression of a wide range of alternative activation markers, could also drive higher levels of bioactive IL-12 production and consequently promoting Th1 type responses. Nevertheless, RELM-alpha expression by DCs downstream of IL4 signaling further promotes IL-10 and IL-13 production, suggesting a more complex and potentially antagonistic relationship between IL-4 induced factors produced by dendritic cells (73).

In keeping with the prevailing theme that helminths induce both Th2 and regulatory immune mechanisms, chronic gastrointestinal helminth infection has also been shown to promote the development of a CD11c^lo^CD103^−^ dendritic cell population that may be important for the expansion of Treg cells during chronic helminth infection (74). Interestingly, these helminth-expanded CD11c^lo^ DCs exhibited poor responsiveness to TLR activation and consequently deficient T cell activating potencies. Instead, naïve T cells stimulated by these CD11c^lo^ DCs were more likely to become Foxp3-positive Tregs. Consistent with a model where distinct DC subsets mediate helminth induction of Th2 and Treg responses, depletion of CD11c^hi^ DCs abrogated Th2 effector responses, while sparing Treg expansion.

The ability of helminths and helminth products to dampen the antigen-presenting and costimulatory functions of dendritic cells and the induction of Tregs may not be the sole mechanism for how these parasites modulate proinflammatory Th1 and Th17 responses. The production of type 2 cytokines by ILC2s, eosinophils, neutrophils and basophils instruct the formation of alternatively activated macrophages, not only at affected tissue sites but also within secondary lymphoid organs. A key difference between these alternatively activated macrophages and their classically activated counterparts is in their alternative metabolism of the amino acid arginine. IL4 induces arginase 1 which results in the formation of ornithine and urea. The elaboration of arginase can result in depletion of this essential amino acid and restrain the activation and function of T cells. T lymphocytes subjected to arginine depletion become blocked in the G1 stage of the cell cycle and subsequently downregulated mTORC1 activity, while mTORC2 mediated cell cycle arrest in these starved T cells (75). In relation to this, it is interesting to note that mTORC1 is required for the generation of Th1 cells and CD8 effector T cells, while mTORC2 is involved in the formation of Th2 cells and CD8 memory T cells (76, 77). Thus, it is likely that alternative macrophage induction, through arginase-modulation of T cell metabolism, may explain why helminths can potently inhibit the generation of heterologous effector Th1 and CD8 CTLs, and instead favor Th2 and memory CD8 T cell responses. In addition, arginine-depletion could also modulate the intrinsic ability of T lymphocytes to signal through the T cell receptor (TCR) by impairing expression of the CD3 zeta chain (78). Thus, by downmodulating the assembly and signaling potency of the TCR, alterations in lymphocyte metabolic pathways and the lack of expression of Th1 promoting chemokines and costimulatory molecules, alternative macrophages can effect, through both lymphocyte intrinsic and extrinsic mechanisms, impose a regime that inhibits proinflammatory effector cell generation and favor Th2 and Treg responses. AAM conversion of both pre-existing tissue-resident macrophages and newly arrived monocyte-derived migrants provide a mechanism to initiate and perpetuate this immunological regime (79, 80) The ability of helminth infection to simultaneously exert immunoregulatory activities on both DCs and macrophages may provide an explanation for how it potently suppresses both differentiation and functional maturation of type 1 effector T cells in the context of *Toxoplasma* coinfection (81). Recent publications have highlighted a requirement for sequential engagement by dendritic cells and macrophages and their production of IL-12 and IFN-γ induced chemokines for optimal type 1 effector cell differentiation (82–84). Thus, helminth immunodulation of the innate immune landscape in both the T cell and the extrafollicular areas of lymphoid organs provides a powerful mechanism to thwart type 1 effector responses. Furthermore, this immunosuppressive mechanism could act dominantly to thwart vaccine-induced protective immunity, despite higher frequencies of memory cells, because the transition from central memory to effector memory or effector cells require costimulatory signals from innate accessory cells (83, 84).

### Perspectives and concluding remarks

**Figure 3:**
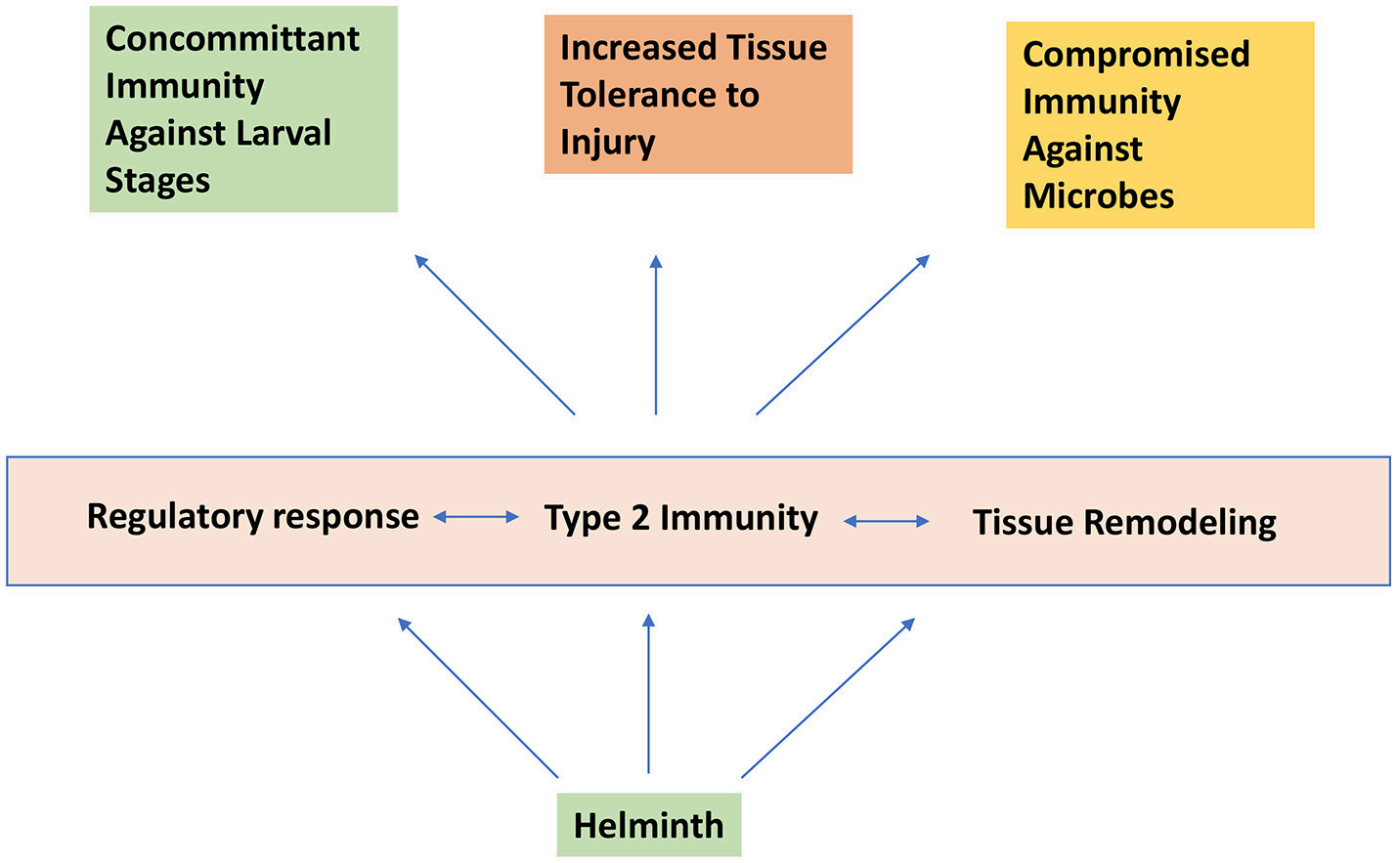
Helminth infection induces concomitant immunity and increased tissue tolerance to injury while promoting compromised immunity to certain microbial agents.

From a teleological perspective, helminth parasites may have coopted the type 2 and Treg response to “optimize” their species-specific host niche (see Figure 3). Besides preventing extensive tissue damage and excessive and overt inflammatory responses, an important consideration would be to limit the overall parasite load that could result in host morbidity and mortality. Because most helminths do not proliferate within the host, a state of concomitant immunity, where the presence of adult worms induces and preserves resistance mechanisms that prevent further infestation by larval stages of the same or even a different helminth organism maybe a useful lens to view the various manifestations of helminth infections. Thus, besides promoting host viability for effective reproduction, tissue remodeling and enhanced immune resistance in the lung and the gastrointestinal tract could be also viewed as a tactic to prevent further colonization. Similarly, alterations in the innate and adaptive immunological landscape in lymphoid tissues may represent a mechanism to perpetuate the changes enforced within host tissues. A striking example of this concept is the recent demonstration that *Trichuris muris* coopts the host microbiota to increase its own fitness and alters the microbiome in such a way that inhibits subsequent rounds of infection (85). It is likely that helminth infection exerts multiple collateral changes in other host tissue systems (e.g., the hemopoietic and neuro-endocrine systems), which may have a profound impact on resistance and/or tolerance to other infectious agents.

In particular, these immune regulatory mechanisms, including products directly produced by the parasite, can modulate the immune response in some cases impairing effective type 1 immunity against microbial pathogens. On the other hand these same tolerance mechanisms, including factors directly enhancing wound healing, may mitigate severity of tissue damage associated with microbial infections, raising the possibility that eradication of helminths may not only enhance resistance but also deleterious effects of type 1 inflammation leading to increased severity of disease. Understanding the multiple mechanisms through which helminths modulate immune responses and promote tissue repair may lead to new and effective targeted treatments to control harmful inflammation associated with microbial pathogens as well as noncommunicable inflammatory diseases.

## Author contributions

All authors listed have made a substantial, direct and intellectual contribution to the work, and approved it for publication.

### Conflict of interest statement

The authors declare that the research was conducted in the absence of any commercial or financial relationships that could be construed as a potential conflict of interest.
